# Comparative morphology, biology and histology of reproductive development in three lines of *Manihot esculenta* Crantz (Euphorbiaceae: Crotonoideae)

**DOI:** 10.1093/aobpla/pls046

**Published:** 2012-11-30

**Authors:** P. I. P. Perera, M. Quintero, B. Dedicova, J. D. J. S. Kularatne, H. Ceballos

**Affiliations:** 1 Cassava Program, Agrobiodiversity Research Area, International Center for Tropical Agriculture, A.A. 6713 Cali, Colombia; 2 Biotechnology Unit, Agrobiodiversity Research Area, International Center for Tropical Agriculture, A.A. 6713 Cali, Colombia; 3 Decision and Policy Analysis Research Area, International Center for Tropical Agriculture, A.A. 6713 Cali, Colombia

## Abstract

**Background and aims:**

Cassava (*Manihot esculenta*), a major food staple in the tropics and subtropics, thrives even in environments undergoing threatening climate change. To satisfy the increasing demand for crop improvement and overcome the limitations of conventional breeding, the introduction of inbreeding techniques such as the production of doubled haploid lines via androgenesis or gynogenesis offers advantages. However, comprehensive studies on cassava flower bud biology or structural development are lacking and precise structural and biological information is a prerequisite to enhance the efficiency of these techniques.

**Methodology:**

The floral biology of three selected cassava lines was studied, focusing on morphology, phenology and pollen biology (quantity, viability and dimorphism). Histological studies were also conducted on microsporogenesis/microgametogenesis and megasporogenesis/megagameto-genesis to generate precise developmental data for these lines.

**Principal results:**

Male and female cyathia have distinct developmental phases. Pollen viability was high during immature stages of plant development; however, pollen mortality was common at later stages. Pollen trimorphism in male gametophytes towards the larger or smaller pollen size, as compared with normal size, was observed. Ten characteristic events were identified in male gametogenesis and six in female gametogenesis that were correlated with flower bud diameter. Male gametophyte diameter at different developmental stages was also determined.

**Conclusions:**

Results indicate that the three lines did not differ significantly, except regarding a few morphological aspects such as plant height, flower colour and number of male cyathia. Pollen grains were initially viable, but viability decreased drastically at later stages of growth. Abnormal meiosis or mitosis triggered pollen trimorphism. The demonstrated sequential events of reproductive development generated valuable information at the cellular level, which will help close the current information gap for cassava improvement via breeding programmes and doubled haploid plant production.

## Abstract (precis)

Cassava flowering with emphasis on flowering pattern, morphology and phenology; pollen biology on viability and dimorphism, and histology on male and female gametophyte development are demonstrated. Reduced pollen viability at anthesis and the existence of pollen tri-morphism are the key findings.

## Introduction

Cassava (*Manihot esculenta*), also known as tapioca, manioc, mandioca and yuca, is one of the most important root crops cultivated in the tropics and subtropics around the world (Raji *et al.* 2009). It belongs to order Malpighiales and family Euphorbiaceae, and its relatives in the Euphorbiaceae family include commercially important plants such as rubber tree (*Hevea brasiliensis*), castor oil plant (*Ricinus comunis*) and ornamental plants such as *Euphorbia* spp. Studies conducted so far indicate that all species have 36 chromosomes that show regular bivalent pairing at meiosis (Jennings and Iglesias 2001; Wang *et al*. 2010).

Cassava is a food staple for >800 million people in South America, the Caribbean, Africa and Asia. It ranks fourth as the source of human energy (Alves 2001), especially in Africa (Groll *et al.* 2001). In addition to its dietary value when consumed by humans in the form of boiled roots, cassava's starchy tuberous roots are a major source of carbohydrates in animal feed and are also used to produce starch and biofuel (FAO 2009). Cassava has been at the forefront as an alternative food source to cereals, which are more expensive and highly variable in price (FAO 2009).

New cassava cultivars have long benefitted both large and small farmers (Henry and Hershey 2002). Crop improvement programmes have aimed to develop high-yielding, disease-resistant and highly nutritive cultivars (Ceballos *et al*. 2010). Cassava is a highly heterozygous species (Bonierbale *et al.* 1997). Being an outcrossed crop, inbreeding may prove difficult and severe depression could occur after inbreeding in both traditional (Pujol *et al.* 2005) and modern production systems (Kawano *et al*. 1978; Rojas *et al.* 2009). However, the introduction of inbreeding in cassava genetic improvement programmes offers several advantages, such as exploitation of recessive traits, decrease of genetic load and production of better-designed hybrids (Rojas *et al.* 2009; Ceballos *et al.* 2010). Inbreeding lines can be obtained by producing doubled haploids, inducing embryogenesis in male gametophytes via androgenesis or female gametophytes via gynogenesis. Techniques need to be developed, however. Precise structural, biological and ontological data on female and male gametophytes are a prerequisite to initiate research to develop these techniques.

Except for two reports, one on the relationship between the stages of anther development and floral bud length (Wang *et al.* 2010) and the other on embryo sac development (Ogburia and Adachi 1996), comprehensive studies on the biology or structural development of flower buds in cassava cultivars are lacking. Little is known about its flowering habit and some clones have never been known to flower. Nevertheless, Rogers and Appan (1973) and Alves (2001) gave a general description of the flowers of this diclinous and monoecious species: the female and male flowers are produced in the same inflorescences (racemes or panicles) on the same plant. However, both citations use erroneous terminology of inflorescence and flower parts. Compared with other plants, flower structures of Euphorbiaceae plants present a remarkable evolution. Cassava flowers are, in fact, apetalous, which means they have no petals or sepals. Female flowers are single and reduced to a pistil that is protected by petal-like bracts. Male flowers are also reduced to a single stamen but, unlike female flowers, they form inflorescences of 10 single-stamen flowers. These inflorescences, known as cyathia (Prenner and Rudall 2007), are also protected by bracts and bracteoles. What are commonly called tepals (i.e. petal-like sepals) are actually bracts.

In this context, an in-depth study of the floral biology of three selected cassava lines, currently used for the development of a protocol for the production of doubled haploids, was undertaken, emphasizing their phenology, morphology and pollen biology (quantity, viability and dimorphism). Histological studies were also conducted that covered the most immature stages of male and female cythia up to anthesis on microsporogenesis/microgametogenesis and megasporogenesis/megagametogenesis to investigate their developmental process at the cellular level. Besides generating precise comprehensive knowledge, the present study aimed to identify any deviation in the aforementioned aspects among the three cassava lines.

## Methods

### Plant materials and study site

Three cassava lines, SM 1219-9, TMS 60444 and HMC 1 (hereinafter referred to as SM, TMS and HMC), were selected for the present study. These lines are currently being used by the Cassava Program of the International Center for Tropical Agriculture (CIAT) in its project ‘Inbreeding in cassava through the production of doubled haploids’. SM is the result of a polycross (open pollination) made in 1988 for which only its female progenitor (CG 1450-4), derived from the Colombian landraces MCOL 1505 and MCOL 1940, is known. TMS originated in Nigeria and has been used as the model line for genetic transformation work (Taylor *et al.* 2004; Liu *et al.* 2007). HMC is a commercial variety released in Colombia from a cross made in 1980. It originated from the irradiation of the landrace MCOL 1438.

All three lines were grown in the field in clay loam soil, pH 7.2, at CIAT headquarters in Cali, Colombia (3°30′N, 76°19′W; 965 m above sea level). During 1993–2010, mean daily temperatures ranged from a maximum of 29.7 ± 0.1 °C to a minimum of 19.2 ± 0.1 °C, with a mean monthly temperature of 23.8 ± 0.1 °C. The relative humidity averaged 78 % and annual rainfall, 936 ± 34 mm. Field management practices consisted of minimum or no application of agrochemicals, biological pest and disease control, mechanical weed control, sprinkler irrigation and application of certain fertilizers.

### Flowering pattern

Forty-nine stem cuttings from each of the three lines were planted in seven subsequent batches, each with seven cuttings. Thirty healthy plants were selected and labelled for the study. Branch height at each flowering was measured, and the dates of first signs of flowering at the main stem, first and second branching events were recorded to determine the duration of time to flowering after planting in the field.

### Inflorescence and flower architecture

To determine the inflorescence structure, 100 racemes from each variety were collected and the number of branches and frequency of male, female and hermaphrodite (if any) cyathia per raceme were counted. Flower colour was observed visually. Ten male cyathia from each line were removed and examined in more detail under a dissecting microscope (SZ2-ILST, Olympus, Tokyo, Japan) to determine stamen structure. Bracts were removed, and the lengths were measured of the anther and filament of stamens excised from both internal and external whorls using an electronic digital calliper (DC 1004, PLG-Discover, China) to determine the structural difference between the two whorls. Filament length was measured as the distance between the filament base and the anther–filament junction.

### Phenology

The anthesis dates of first and last male and female cyathia in the racemes of each plant were recorded to determine the male and female phases. In addition, 30 male and female cyathia from each of the above selected plants were labelled, and the anthesis and abscission dates were recorded to determine the life span of the two flower types.

### Pollen grains

The number of pollen grains per anther was determined by counting the total of pollen grains released onto a glass slide. Counts were based on two anthers each of both internal and external whorls of 100 randomly selected male cyathia. Fresh anthers were gently squashed in a water drop on a glass slide, covered with a 12-mm-square cover slip and then the pollen grains were counted.

Pollen viability was assessed using modified Alexander's staining (Peterson *et al.* 2010) and the KI/I2KI/Lugol's (Bolat and Pirlak 1999) method. A total of 100 freshly collected male cyathia (containing mature pollen grains), 3.2 mm in diameter, from each line were fixed in Carnoy's fixative solution (ethanol : glacial acetic acid, 3 : 1) until used. The average number of viable pollen grains was determined by counting the dark purple grains using modified Alexander's staining and the blackish orange grains using KI in three microscopic fields for each sample. The status of viability in different branching levels was also assessed by collecting 100 male cyathia from each ramification.

The occurrence of pollen dimorphism in the three lines was studied by measuring the diameter of the pollen grains extracted from a single anther of each of the 100 male cyathia, 3.2 mm in diameter. As indicated previously, the collected cyathia were fixed until used. The pollen grains were released onto a glass slide by pressing the anther, and the diameter was measured in 20 randomly selected pollen grains under the light microscope (Laborlux D, Leitz, Wetzlar, Germany) at a magnification of ×20. Based on the generated data, additional studies were undertaken to determine the occurrence and frequency, if any, of pollen dimorphism. The presence of tetrads was also evaluated to determine their relationship with the occurrence of pollen dimorphism.

To determine whether the dimorphism was towards the larger or smaller pollen size as compared with the normal size category, the diameter data of 100 anthers were filtered and anthers were divided into two categories: anthers with only normal-sized pollen grains and anthers with dimorphic pollen grains. Using the data of the normal size category, the diameter range of normal pollen grains was determined for each line and then used as the basis to screen the data of dimorphic anthers to determine whether the deviation is towards the larger or smaller grain size. The number of dimorphic anthers was also counted.

### Histology

A histological analysis of sporophyte and gametophyte development in male and female cyathia was conducted to compare the stages at which the sequential developmental events take place in the cyathia of the three lines under study. To correlate male bud size with microsporogenesis and microgametogenesis, from three to six male cyathia of each line, at successive developmental stages, were randomly collected from the field-grown plants for each of the following size categories and measured with the electronic digital calliper: 0.1–2.0 mm at 0.5-mm intervals, 2–4 mm at 0.1-mm intervals and ≥4 mm at 0.5-mm intervals. Similarly, to correlate female cyathium size with megasporogenesis and megagametogenesis ontogeny, the following size categories were analysed: 0.5–2 mm at 0.5-mm intervals, 2–4 mm at 1-mm intervals and 4–11 mm at 0.5-mm intervals. From 224 to 240 male and female cyathia, representing a series of all possible sizes up to anthesis, were sectioned from each line. Only the anthers or ovaries were extracted from the later stages, whereas whole cyathia were used from the initial developmental stages.

To ensure freshness of materials, floral buds were kept in a humid box until categorized and fixed. Just after sampling, materials were fixed in FAA (50 % ethanol + 10 % formaldehyde + glacial acetic acid, 18 : 1 : 1) for 72 h, with the solution being changed twice per day. The samples were then dehydrated through a graded ethanol series (30, 50, 70, 95 and 100 %, v/v), being left for 2 h in each solution. The samples were further dehydrated by transferring them to 100 % butanol for 48 h. After impregnation, the samples were embedded in resin, Technovit 8100^®^ (Heraeus Kulzer GmbH, Wehrheim, Germany), and allowed to polymerize for 2 h at room temperature. Sections (3.5 μm thick) were obtained using a rotary microtome (Histostat, Reichert Scientific Instruments, NY, USA) and then double-stained with periodic acid Schiff's (Sigma Aldrich, Lyon, France) for starch and protein-specific naphthol blue black. All the sections were studied under the light microscope (DM500, Leica GmbH, Wetzlar, Germany) and photographed with an attached camera (ICC50HD, Leica GmbH).

Specific microgametogenesis stages were characterized based on the histological micrographs. The diameter of the male gametophyte, including the sporodermis at each microgametogenesis stage, was determined by measuring the diameter of 100 grains with Image-Pro Plus^®^ software (Media Cybernetics, MD, USA). The number of non-viable pollen grains at different stages of anther development was recorded to correlate pollen viability with pollen developmental stage.

### Statistical analysis

The mean and standard error were calculated for all variables measured. Variations among the three lines were assessed by analysis of variance using SAS statistical package (SAS, 1999) where applicable. The least significant difference test at *P* = 0 ± 0.05 was used to compare the means if there was a significant difference among the variables.

## Results

### Flowering pattern

**Fig. 1:**
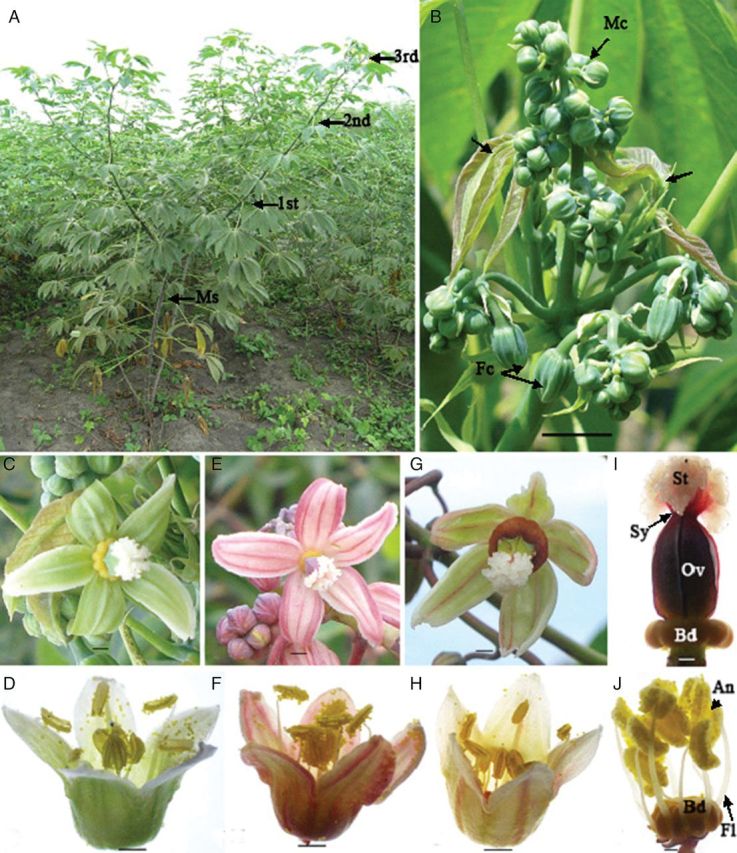
**Morphological aspects of *M. esculenta* inflorescence.** (A) Branching/ramification of SM 1219-9, which is typical of branching type cassava genotypes. Note the branching pattern of main stem (Ms), 1st, 2nd and 3rd. (B) Flowering at the terminating branch. Note that the development of the next branching level (arrows) simultaneously at the flowering end and different racemes bearing female (Fc) and male (Mc) cyathia (scale bar = 17.5 mm). (C) Female cyathium (a single female flower reduced to one gynoecium consisted of five nectar glands covered by five petal-like bracts) of SM 1219-9 (scale bar = 1.25 mm). (D) Male cyathium (inflorescence composed of 10 male flowers reduced to 10 stamens covered by five petal-like bracts) of SM 1219-9 (scale bar = 1 mm). (E) Female cyathium of HMC 1 (scale bar = 1.25 mm). (F) Male cyathium of HMC 1 (scale bar = 2 mm). (G) Female cyathium of TMS 60444 (scale bar = 1.35 mm). (H) Male cyathium of TMS 60444 (scale bar = 2 mm). (I) Gynoecium. Note the ovary (Ov) with short style (Sy) and three-lobed stigma (St) (scale bar = 1 mm). (J) Androecium. Note the filaments (Fl) with dosifixed anthers (An) arise from the basal disk (Bd) (scale bar = 1 mm).

The vegetative branching system common for studied cassava lines is shown in Fig. 1A. The main stem of each tested line produced a weak inflorescence bearing a few unfertile male cyathia. True flowering was observed only at the first branching level, giving rise to healthy racemes bearing both male and female cyathia. Inflorescence and ramification initiation occur simultaneously at the top of each branching level (Fig. 1B). The lines differ significantly in branch heights (*P* < 0.001; Table 1).

**Table 1:** Morphological aspects of *M. esculenta* lines SM 1219-1, TMS 660444 and HMC 1.

Observed character	Details	SM 1219-9	TMS 660444	HMC 1
Branch height at flowering (cm)	Main stem	122.2 (4.2)*^a^	46.2 (2.2)^†^	68.0 (3.3)^†^
	First branch	177.5 (4.9)	88.3 (3.1)	112.2 (3.9)
	Second branch	247.0 (2.9)	178.0 (4.3)	148.5 (3.8)
Flowering phenology (days)	Initiation of inflorescence after field planting	147.0 (1.1)	137.6 (1.7)	135.7 (2.8)
	Opening of first female cyathium	25.1 (1.1)	25.8 (0.9)	24.0 (1.8)
	Female phase	1.0 (0.2)	1.0 (0.2)	1.0 (0.3)
	Opening of first male cyathium	28.5 (0.7)	29.5 (0.8)	29.9 (1.3)
	Male phase	12.8 (0.7)	13.8 (0.3)	9.0 (0.3)
Flower life span (days)	Male cyathium	11.8 (0.4)	11.4 (0.2)	9.8 (0.2)
	Female cyathium	9.6 (0.4)	9.5 (0.3)	9.0 (0.5)
Inflorescence architecture (number)	Branches	4.9 (0.1)	4.3 (0.3)	3.9 (0.5)
	Male cyathia	139.7 (7.3)^‡^	49.5 (2.8)^§^	26.1 (1.7)^§^
	Female cyathia	6.9 (0.2)	6.5 (0.2)	4.9 (0.2)
	Hermaphrodite cyathia	0.04 (0.02)	0.03 (0.02)	0.2 (0.04)
Flower morphology	Colour	Green	Yellowish green	Reddish green
	Number of anthers per male cyathia	10	10	10
	Number of ovules per female cyathia	3	3	3
	Pollen to ovule ratio	606	616	577
Stamen size (length in mm) in different whorls	External filament	6.63 (0.05)	7.27 (0.1)	7.09 (0.14)
	Internal filament	3.61 (0.04)	4.9 (0.17)	4.43 (0.11)
	External anther	2.49 (0.02)	2.52 (0.03)	2.40 (0.03)
	Internal anther	2.41 (0.02)	2.42 (0.02)	2.39 (0.03)
Pollen quantity	External anther	185.2 (2.3)	190.1 (2.3)	176.8 (2.4)
	Internal anther	178.0 (2.1)	179.58 (2.5)	167.6 (2.6)
	Average number of pollen grains per flower	1815.8 (29.2)	1848.6 (32.8)	1722.4 (34.1)

Different symbols within a row indicate significantly different values at *P* < 0.001.

^a^Values averaged and standard error in parentheses.

### Inflorescence and flower architecture

Details of the structural morphology of the inflorescences and individual cyathia of each line are summarized in Table 1. The cassava raceme (Fig. 1B) consisted of an average of 4–5 branches, with only small differences among the three lines. These branches were further divided into primary sub-branches that gave rise to secondary sub-branches. Cyathia were concentrated on the apical part of the branches. Normally, two female cyathia with long petiole were located at the base of the flower-bearing area of each main branch. The male cyathia were concentrated on the apical part of the branches having a very short petiole. The number of male cyathia per raceme differed significantly among the three lines evaluated (*P* < 0.001; Table 1). Line SM contained the highest average number of male cyathia per inflorescence (≈140), whereas TMS averaged only 50 and HMC, 26 (Table 1). The average number of female cyathia per inflorescence was 6.1 %, ranging from 4.9 % for HMC to 6.9 % for SM. Hermaphrodite flowers were observed rarely, with an average of 0.2–0.03 %, with HMC presenting the maximum value and TMS the minimum.

There were distinct differences in flower colour among the three lines evaluated, ranging from light green in SM (Fig. 1C and D) to pink with red stripes in HMC (Fig. 1E and F) to greenish yellow with red stripes in TMS (Fig. 1G and H). Male and female cyathia were covered with five bracts, which are solitary in female cyathia (Fig. 1C, E and G) and partially fused in male cyathia (Fig. 1D, F and H). Each female cyathium contained a single ovary with a very short style and three-lobed stigmas that developed on a special structure called the basal disk, composed of fused honey glands in the female cyathium (Fig. 1I) and incompletely fused in the male cyathium. Various pistil parts differ in colour among the three lines. Each male cyathium contained 10 staminate flowers with a single stamen forming two whorls (Fig. 1J). The individual filament developed from the deep basal disk holds a dorsifixed versatile anther. The external stamen filament is longer than the internal one, creating enough space for the 10 anthers to fit into the small space within the cyathium bud. The internal filament (ranging from 3.6 to 4.9 mm) was ∼75 % the size of the external one (ranging from 6.6 to 7.3 mm), with SM presenting the shortest filament and TMS the longest (Table 1). In all three lines, the external anther was longer than the internal one, ranging from 0.01 mm in HMC to 0.1 mm in TMS.

### Phenology

From the onset of inflorescence primordia, it took ∼24–26 days for the first female cyathium to open and 29–30 days for the first male cyathium, regardless of the line (Table 1). Female cyathia always bloomed before neighbouring male cyathia, with a 4-day difference between the anthesis of the two flower types (Table 1). All female cyathia of a raceme opened the same day, whereas male cyathia opened over a 12-day period. The shortest male phase (9 days) was observed in HMC, whereas the other two lines showed almost identical male phases in terms of length: 12.8 days for SM and 13.8 for TMS (Table 1). The average life span of individual male cyathia was 10–12 days, while that of female cyathia was ∼9–10 days (Table 1).

### Pollen grains

The mean number of pollen grains per male cyathium was 1816 for SM, 1849 for TMS and 1722 for HMC (Table 1). Pollen count between whorls revealed that the quantity of pollen of the external whorl was always greater than that of the internal whorl.

**Fig. 2:**
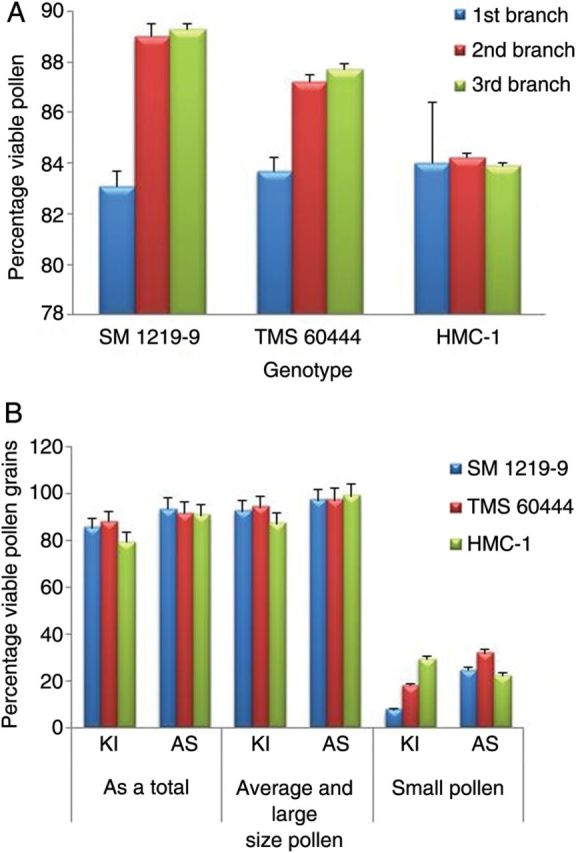
**Pollen viability in the three *M. esculenta* genotypes evaluated.** (A) Average pollen viability at different branching levels (first, second and third). (B) Pollen grain viability with two staining methods.

No significant difference was observed between the different ramifications (first and second; Fig. 2A), while all the flowers produced on the main stem were sterile. The viability of pollen grains (%) determined by modified Alexander's staining was comparable with that determined by the KI staining method (Fig. 2B). No significant difference was observed among the lines with either staining method used. Further analysis revealed that the percentage of viability was higher in the normal- and large-sized pollen grains as compared with the smaller ones: 85.3, 92.4 and 7.8 % for SM; 88.1, 94.2 and 18.0 % for TMS; and 79.3, 87.1 and 29.2 % for HMC (Fig. 2B). In all three lines, most of the smaller pollen grains were non-viable (Fig. 2B).

**Fig. 3:**
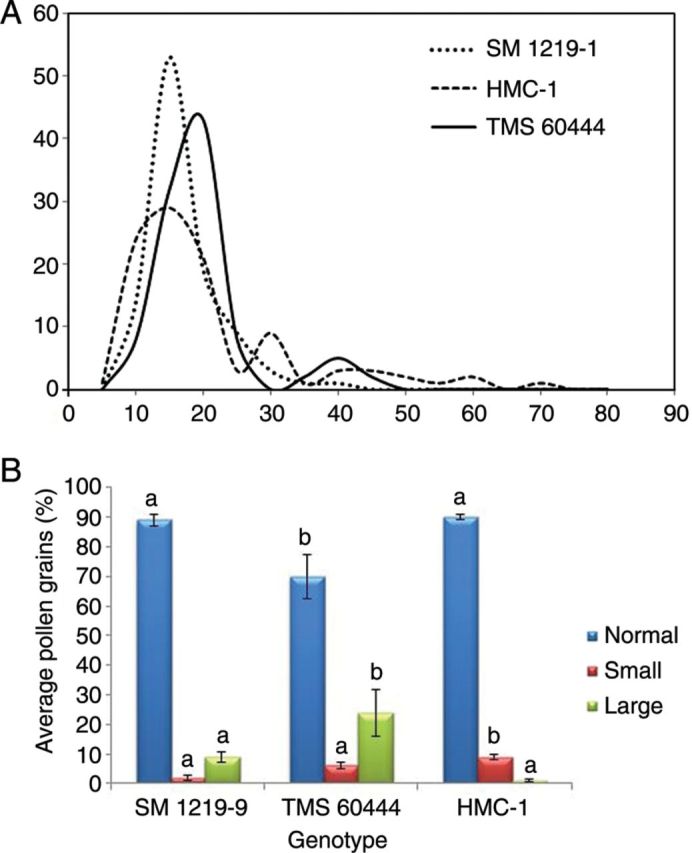
**Pollen dimorphism in *M. esculenta* genotypes evaluated.** (A) Frequency of pollen grains in each category based on the difference in grain diameter for the three genotypes. Note that the normal difference ranges between 5 and 30 µm. A difference of over 30 µm was taken as dimorphic pollen grains. (B) Average number of pollen grains in the different size categories (larger and smaller size as compared with normal size) within the anthers showing pollen dimorphism. Different letters above each column indicate significantly different values at *P* < 0.05.

The results clearly indicated the existence of pollen dimorphism in all three lines studied. The pollen grains were spherical in all lines with a thick exine. The differences between the maximum (*D*_max_) and minimum (*D*_min_) diameters of the pollen grains were established within individual anthers. Frequency distributions for differences in pollen sizes were then established for the three lines being evaluated (Fig. 3A). The graph clearly shows a higher frequency of size differences in pollen grains fell between 5 and 30 µm. Therefore, 30 µm was set as the maximum acceptable difference in pollen size within an anther. Beyond this figure, differences in pollen size were considered a dimorphism. Student's *t*-tests were also conducted and, based on the results, the total size difference was divided into two groups at different cut-off points. Test results also suggested a demarcation of the size difference for dimorphism at 30 µm (*P* < 0.001). Line HMC showed the greatest dimorphism (22 % of its anthers with pollen size differences >30 µm) as compared with the results for TMS (9 %) and SM (5 %) (Table 1).

Results indicated that the anthers of SM and HMC contained ∼10 % dimorphic pollen grains, whereas TMS contained 30 %. Overall, 90 % of the pollen grains of lines SM and HMC were of normal size as compared with only 70 % in the case of TMS (Fig. 3B). The frequency of large pollen grains was significantly higher in TMS as compared with SM, but was almost negligible compared with HMC (Fig. 3B).

**Fig. 4:**
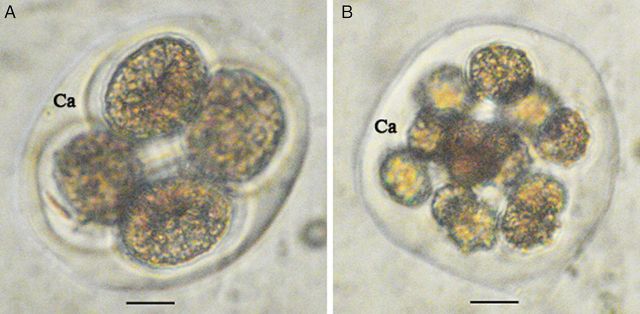
**Morphological aspect of *M. esculenta* microspores within the callose (Ca) matrix.** (A) Normal tetrad (scale bar = 12.3 µm). (B) After undergoing multiple cycles of mitosis (scale bar = 14.4 µm).

Additional observations revealed that, after undergoing meiosis and first mitosis, most of the time the callose matrix contained the four cells (Fig. 4A) typical of this development stage. However, as shown in Fig. 4B, in a few cases the number of cells counted was higher, either 8 or 16, or lower, being 2 (or dyads) (data not shown), than the expected number.

### Histology

The following descriptions are similar for all the lines studied and only distinctive features or differences are specifically mentioned. A sequential developmental event in male and female gametophytes was clearly identified in the histological analysis. Ten events in male gametogenesis and six in female gametogenesis were correlated with the relevant bud diameter size category (Table 2). Table 3 summarizes the diameters of the male gametophytes of the three lines at different developmental stages.

**Table 2:** Correlation of the diameter of the flower bud with the events in male and female gametogenesis in three *M. esculenta* lines.

Sequential events occurring in the gametogenesis pathway	Diameter (mm) of the cyathia at which the respective event occurred		
	SM 1219-9	TMS 60444	HMC 1
Microsporogenesis and microgametogenesis			
Formation of microspore mother cell (MMC)	0.1–0.5	0.1–0.5	0.1–0.5
Meiosis and formation of tetrads	1.5–2.0	1.5–2.0	1.5–2.0
Callose matrix degeneration and exposure of the microspores	2.0–2.1	2.0–2.1	2.0–2.1
Formation of early uninucleate microspores	2.0–2.1	2.0–2.1	2.1–2.2
Formation of mid-uninucleate microspores	2.1–2.2	2.1–2.2	2.2–2.3
Formation of late uninucleate microspores	2.2–2.3	2.5–2.6	2.3–2.4
Formation of binucleate microspores	2.3–2.5	3.1–3.2	2.5–2.6
Beginning of amyloplast accumulation	2.7–2.8	3.3–3.4	2.7–2.8
Termination of amyloplast accumulation	5.5–6.0	6.5–7.0	3.9–4.0
Pollen grain mortality	6.0	7.0	4.0
Megasporogenesis and megagametogenesis			
Carpel differentiation in the ovary	0.5–1.0	0.5–1.0	0.5–1.0
Differentiation of the ovule	1.0–1.5	1.0–1.5	1.0–1.5
Archesporium development	1.0–1.5	1.5–2.0	1.5–2.0
Megasporogenesis of the MMC	2.5–2.6	2.2–2.3	2.3–2.4
Embryo sac formation	4.5–5.0	6.0–6.5	5.0–5.5
Starch accumulation in the nucellus	7.0	8.5	6.5

**Table 3:** Correlation of the microspore developmental stage with male gametogenesis events; average diameters with standard errors are given for each stage.

Microspore developmental stage	SM 1219-9	TMS 60444	HMC 1
Freshly released microspores from tetrads	50.7 (0.5)^a^	64.3 (0.4)	54.5 (0.3)
Early uninucleate microspores	79.5 (1.0)	91.7 (0.5)	71.5 (0.8)
Mid-uninucleate microspores	100.6 (0.6)	103.7 (0.7)	93.1 (0.5)
Late uninucleate microspores	125.2 (0.5)	124.1 (0.8)	119.9 (0.5)
Early bicellular	131.7 (0.7)	133.1 (0.5)	129.3 (0.4)
Early amyloplast accumulation	140.8 (0.3)	164.1 (0.9)	141.5 (0.6)
Late amyloplast accumulation	160.6 (0.3)	174.8 (1.0)	163.1 (0.3)

^a^Values averaged and standard error in parentheses.

#### Microsporogenesis and microgametogenesis

##### Formation of the microspore mother cell

**Fig. 5:**
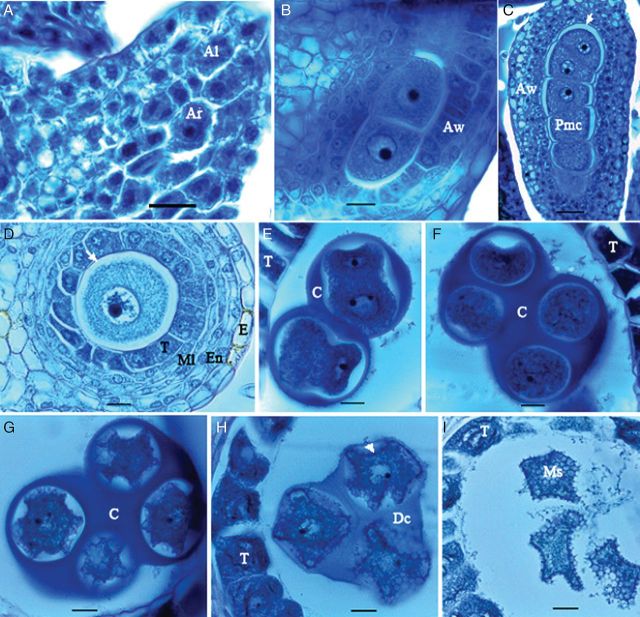
**Microsporogenesis in *M. esculenta.*** (A) Formation of archesporium (Ar). Note the larger cell size and the prominent nucleus as compared with the surrounding cells of the anther locule (Al). (B) Archesporium undergoing mitosis to produce meiocytes. Note the differentiating neighbouring cells in the different cell layers of the anther wall (Aw) in the longitudinal section (LS). (C) Meiocyte/MMC/Pmc (pollen mother cell) strands produced in the LS of the anther. Note the thin, pink-stained membrane (white arrow) of callose (C) enclosing the meiocytes. (D) Cross-section of the anther lobe, differentiated epidermis (E), endothecium (En), middle layers (Ml) and tapetum (T). The arrow indicates the callose. (E) Microspore mother cells after meiosis. (F) Early tetrad. (G) Late tetrad containing microspores with uneven periphery. (H) Degeneration of the callose (Dc) matrix. Note the initial synthesis of exine on the microspore surface (arrow). (I) Microspores freed to the pollen sac (scale bars A–I except C = 13.7 µm; C = 30.7 µm).

Each anther had four microsporangia arranged in two thecae. Observations revealed that the reproductive organ differentiation in male cyathia took place at a very early stage (<0.5 mm) of flower development. One cell in the centre of each microsporangium enlarged with a prominent darkly stained nucleus and formed the meiocyte (Fig. 5A). By dividing this meiocyte (Fig. 5B), a strand of microspore mother cell (MMC; Fig. 5C) was formed. The MMC was initially surrounded by uniform cells containing active nuclei with a high nucleus-to-cytoplasm ratio; however, neighbouring cells soon started to differentiate, forming different cell layers of epidermis, endothecium, middle layers (three in SM and two in both TMS and HMC) and tapetum cells (Fig. 5D). With further development of the anther, a space formed between the tapetum and MMC (Fig. 5D). In this space, a pink-stained membrane represented the callose surrounding the MMC (Fig. 5C and D). The MMC had a large nucleus with a conspicuous nucleolus and dense cytoplasm.

##### Meiosis and formation of tetrads

The MMC strand was partially separated by the callose surrounding individual MMCs (Fig. 5E). By undergoing meiosis and first mitosis, cytokinesis took place and formed the tetrahedral tetrad (Fig. 5E and F). Different maturity stages of tetrads were observed in the different flowers. Enclosed microspores in immature tetrads were round, small and centrally located in the cavity. The cells were polarized to the centre of the callose matrix, forming an outward curve. Multiple curves occurred in the following stage, forming a star-shaped microspore with an uneven periphery (Fig. 5G). A small, darkly stained nucleus was present in the centre of these microspores at each stage. TMS differed from the other two lines because it contained highly contracted cytoplasm within the MMC. The initial synthesis of exine was indicated by the black-coloured deposits that could be identified at the periphery of multi-curved tetrads at later stages (Fig. 5H). The callose matrix then began to degenerate, exposing the microspores to the anther locule (Fig. 5I).

##### Disintegration of the callose wall and freeing of microspores

Callose matrices then fully degenerated while the freed microspores still maintained the uneven periphery but with reduced curves (Fig. 5I). There were no differences between the microspore cytoplasm at this stage and at the previous stage; however, at this stage, blue-stained fibrillar substances filled the locule between freed microspores (Fig. 5I).

##### Formation of early uninucleate microspores

**Fig. 6:**
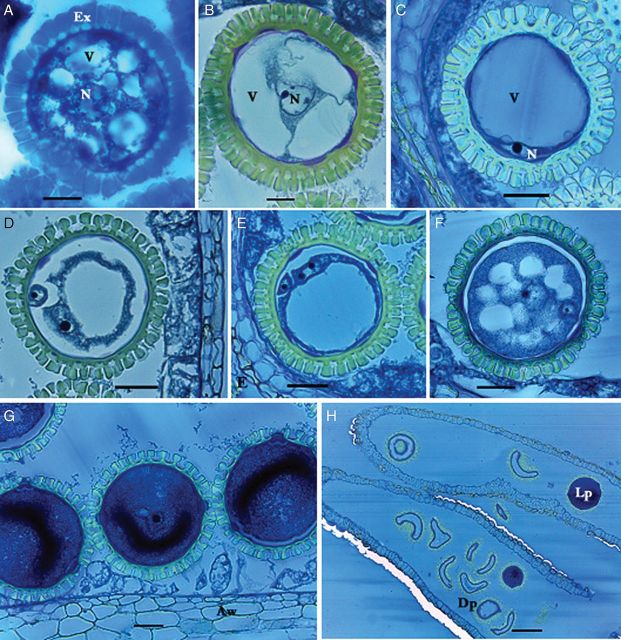
**Microgametogenesis in *M. esculenta.*** (A) Early uninucleate microspore. Note dozens of vacuoles (V) with central nucleus (N) forming a star-shape microspore enclosed in mass-synthesized exine (Ex). (B) Mid-uninucleate gametophyte with three vacuoles. Note the centrally located nucleus. (C) Late uninucleate gametophyte with single vacuole. Note the cytoplasm containing nucleus is restricted mostly to the cell periphery. (D) Binucleate gametophyte. A small generative nucleus is located at the periphery whereas the large vegetative nucleus is moving towards the centre along with the cytoplasm. (E) Binucleate gametophyte. Note the vegetative nucleus with two prominent nucleoli. (F) Early deposition of protein and starch in the cytoplasm. Note the generative cell is enclosed in the large vegetative cell. The large vacuole is replaced by the cytoplasm, forming small vacuoles. (G) Male gametophytes completely filled by dense cytoplasm. (H) Mature pollen sac. Note the numerous deformed dead pollen (Dp) grains and live ones (Lp) with dense cytoplasm in the anthers (scale bars A–B = 13.7 µm; C–G = 30.7 µm; H = 126 µm).

The central nucleus in these microspores became larger, with a prominent nucleolus, while the cells started to take on a round shape (Fig. 6A). Most of the exine synthesis had occurred within this stage, indicating a rapid event. Small vacuoles forming numerous cytoplasmic strands created a star-shaped microspore. Fibrillar substances were present in the anther locule. The aperture of the future pollen grains and intine was also formed.

##### Formation of mid-uninucleate microspores

With the increase in the size of immature pollen grains, two or three large vacuoles formed by merging dozens of small ones (Fig. 6B). The nucleus was centrally located and stained dark. Then the nucleus started to migrate towards the periphery with the moving cytoplasm. Fibrillar substances were still present.

##### Formation of late uninucleate microspores

The pollen grains were then completely filled by a single large vacuole, restricting the cytoplasm to just the periphery (Fig. 6C). At a later stage of this event, the nucleus contained two or three prominent nucleoli, anticipating the upcoming occurrence of mitosis. Pollen grains were tightly packed in the locule, indicating a rapid growth of the pollen grains as compared with the anther locule.

##### Formation of binucleate microspores

Two nuclei with prominent nucleoli were initially located at the periphery of the pollen grain (Fig. 6D and E). One moved towards the centre, filling the cytoplasm, while the other lagged behind (Fig. 6F).

##### Early accumulation of amyloplasts

The single vacuole was gradually replaced by the cytoplasm, producing a blue staining from the periphery to the centre of the pollen grains (Fig. 6F). The vegetative nucleus also migrated towards the centre, along with the cytoplasm. After the starch and proteins were deposited, several small vacuoles formed, again creating a star shape. The cytoplasm, however, was considerably denser than in the previous stage. The central nucleus consisted of a prominent large nucleolus. Throughout this process, fibrillar substances were interspaced with the pollen grains within the anther locule.

##### Late accumulation of amyloplasts

Pollen grains were completely filled with protein and starch accumulates (Fig. 6G). The intensity of the colour of the cytoplasm was considerably greater, and the vegetative nucleus was hardly visible. The small generative cell was surrounded by the large vegetative cell, the exine particles expanded, the anther wall was restricted to the lignified epidermis, and the endothecium and the tapetum disappeared completely. During this event, the mortality rate of pollen grains averaged 4.2 % for SM, 4.5 % for TMS and 13.7 % for HMC.

##### Pollen grain mortality

Small vacuoles reappeared and then expanded to form large vacuoles. In some pollen grains the vacuole was ∼75 % of the cytoplasm volume. The cytoplasm contracted and migrated to one corner, keeping a space between the intine and cytoplasm, the cytoplasm emerging through the broken exine. Most of the cells were empty, changing their shape from round to curved (Fig. 6H). This was a characteristic feature of pollen grains in later stages of maturity. The frequency of these malformed empty pollen grains increased to 90 % throughout subsequent development. This event was observed to occur starting from an average grain diameter of 6 mm in SM, 7 mm in TMS and 4 mm in HMC.

#### Megasporogenesis and megagametogenesis

##### Carpel differentiation in the ovary

The carpels were already differentiated in the smallest isolated female cyathia (0.5–1.0 mm). Two cell lines with periclinal cell division were prominent along the inner surface of the carpel locule, being observed throughout megasporogenesis through late megagametogenesis. Rudimentary stamen initials could be observed in the basal disk. Vascular bundles developed from the petiole towards the ovary. The gynoecium was comprised of three carpels and locules, differentiated in the basal part of the ovary.

##### Ovule differentiation

**Fig. 7:**
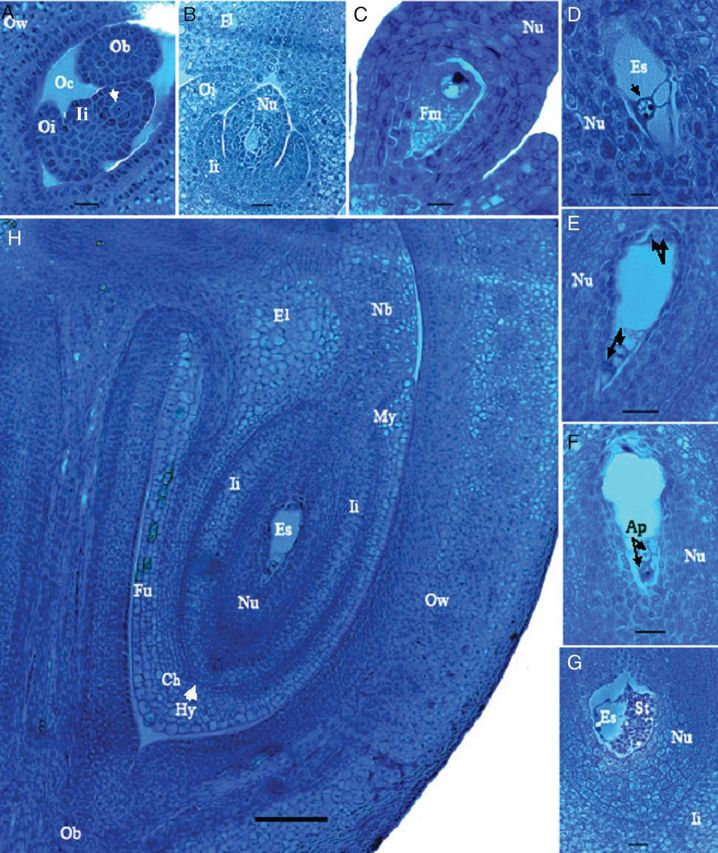
**Megagametogenesis in *M. esculenta.*** (A) Differentiation of archesporium (arrow), inner integument (Ii) and outer integument (Oi) of the ovule. Note a part of the obturator (Ob) in the ovarian cavity (Oc) and the prominent two cell layers in the ovary wall (Ow). (B) Further development of the ovule. Note the elaiosome (El) developed from the upper end of the outer integument forming a neck. The elongating nucellar (Nu) that forms the nucellar beak will grow through the space of the two elaiosomes. (C) Functional megaspore (Fm) developed by the division of the archesporium. (D) Two nuclei in the embryo sac (Es) after meiosis. (E) Four nuclei, two nuclei (arrows) at each end of the embryo sac. (F) Antipodals (Ap) at the chalazal end of the embryo sac. (G) Mature embryo sac with starch (St) deposition in the nucellus, which extends to the inner integument. (H) A complete ovule within one ovary cavity of one locule. Note the nucellar beak (Nb) developed through the elaiosomes at the micopyle (My) end and the hypostasis (Hy, with arrows) at the chalazal (Ch) end of the ovule. The funiculus (Fu) with vascular bundles is parallel to the nucellus and the columella.

The development of the ovule primordia and the obturator occurred simultaneously. Ovule primordia emerged from the columella of each carpel ovary as small protuberances and then curved upwards, showing initials of the integument from epidermal and subdermal cells. The inner integument began as an outgrowth from the dermal cells of the nucellus, whereas the outer integuments had already developed below the inner integument (Fig. 7A).

##### Archesporium development

A single subdermal nucellar cell located from three to five cell layers below the apex of the epidermis enlarged and displayed a prominent nucleus representing the archesporial cell (Fig. 7A). Periclinal divisions in the integuments increased the number of cell layers, and anticlinal divisions were responsible for the growth parallel to the nucellus. The outer integument shortly overgrew the inner integument and fully enclosed the nucellus and inner integument, forming a hood-like elaiosome at the top, with a neck forming just at the top level of the nucellus (Fig. 7B).

##### Megasporogenesis

After meiosis the archesporial cell formed tetrads with the functional megaspore (Fig. 7C) located at the micropylar end. Meanwhile, the nucellus continued to grow, giving rise to a special structure called the nucellar beak that developed through the inner integument and elaiosome. At this stage the funiculus was parallel to the nucellus, forming an upward ovule.

##### Megagametogenesis

The functional megaspore undergoes three successive mitotic divisions to produce an eight-nucleate megagametophyte. The first two nuclei migrate to the poles of the embryo sac cavity (Fig. 7D), and divide to form a four-nucleate embryo sac (Fig. 7E). The fully developed embryo sac contained nuclei, an egg cell, two synergids (collectively called the egg apparatus) at the micropylar end, three antipodals at the chalazal end and two central polar nuclei. In the present study, however, the full egg apparatus could not be observed in the mature embryo sac. The embryo sac expanded during the entire process. In the mature stages only antipodals could be identified (Fig. 7F). In brief, all three lines contained a monosporic, initially seven-celled, eight-nucleate polygonum-type embryo sac.

##### Starch accumulation in the nucellus

With the formation of the complete embryo sac, a massive accumulation of amyloplasts occurred within the embryo sac as well as in nucellus cells at the chalazal end (Fig. 7G). This accumulation subsequently expanded through the nucellus up to the inner integument. Meanwhile, the embryo sac expanded and, at later developmental stages, the nuclei could seldom be observed. At anthesis, the adjoining nucellus cells were enlarged and loosely arranged, allowing space for pollen tube germination. Vascular bundles developed in the funiculus, followed by outer integument (Fig. 7H).

## Discussion

In cassava, flowering induces the formation of reproductive branches. However, some genotypes never flower and therefore never branch (Alves 2001). The selected lines have profuse flowering, which is desirable for ongoing research to develop protocols for the production of doubled haploids in cassava. Stems normally branch in a dichotomous or trichotomous manner, with the branching point exhibiting a terminal inflorescence (Nassar 2000). However, regardless of the line, in this study the main stems stopped their growth with the first flowering event, which typically produced sterile flowers. The first flowering event originated the first branching event, allowing the plant to continue its vegetative growth. The apical growth of first-level branches eventually produces an inflorescence with fully fertile and viable flowers capable of producing fruits and seeds. This pattern may continue for several other flowering events within the same year.

The evaluated lines gave rise to healthy racemes on the first branches within 4–5 months after field planting. Byrne (1984) stated that the time interval from planting to flowering depends on both genotype and prevailing environmental conditions, and may vary from 1 to >24 months. The evaluated lines differed significantly in height at the time of flowering, and these differences could be attributed mainly to internode length (data not shown). All three lines started flowering approximately at the same time after field planting.

Cassava flowers are unisexual, reducing the androecium in female cyathia and the gynoecium in male cyathia. However, the presence of hermaphrodite cyathia (Table 1) indicates that the evolution from complete to unisexual state is not yet complete. Histological studies confirm this hypothesis, showing the reduced androecium (staminodes) submerged in the basal disk of the female cyathium. While the number of female cyathia ranged from 5 to 7, the number of male cyathia fluctuated between 26 and 140, being lowest in HMC and highest in SM. According to Kelly *et al.* (2002), the high number of male cyathia could enhance plant fitness in at least two ways: increased attractiveness to pollinators, thus ensuring that flowers receive sufficient pollen, or enhanced donation of pollen to fertilize flowers of other plants. Since cassava pollination is etomophilous, both arguments are acceptable.

Female cyathia opened 3–6 days before male ones and therefore all three lines show protogyny. Study results clearly indicated distinct male and female phases. Within an inflorescence, all female cyathia bloomed the same day, whereas the male cyathia opened over a period of 9–14 days. These findings do not agree with those reported by Jennings and Iglesias (2001), who found that female cyathia opened 10–14 days before male cyathia on the same inflorescence. The distinct sexual phases within the inflorescences clearly favour cross-pollination (xenogamy) given that cassava is a highly heterozygous species (Kawano *et al.* 1987). However, self-fertilization can occur because the blooming of male and female cyathia on different branches or on different plants of the same genotype (geitonogamy) can occur simultaneously (Jennings and Iglesias 2001) and this was not evaluated in the present study. Other reports indicate that in some cassava lines, such as TMS 89/00037-17, the female cyathia rarely opened when pollination occurred under natural conditions (Hahn *et al.* 1992).

The quantity and quality of pollen produced by the male flowers play an important role for the success of pollination where the pollen output is related to fruit and seed setting (Kulloli *et al*. 2009). The significantly lower number of male cyathia in lines TMS and HMC contributes to their low pollen grain yield, which in turn affects the percentage of seed set in crosses with these lines. Study results indicate that the mean number of pollen grains per flower was 1796 and the mean number of ovules per flower 3. This higher pollen-to-ovule ratio also suggests the cross-pollination nature of these lines (Kulloli *et al.* 2009). Vasek and Weng (1988) mentioned that the evolution of inbreeding in flowering plants has commonly been accompanied by a decrease in the number of pollen grains produced per flower.

Pollen quality is often equated with pollen viability, i.e. the proportion of viable pollen grains (Kelly *et al*. 2002). Some plants had high pollen production but the pollen was of very low quality, suggesting that pollen viability may have a simpler mechanism of genetic determination (Dufay *et al*. 2008). However, there are a number of non-genetic causes of pollen non-viability, including pollen age and physical factors such as temperature and humidity (Kelly *et al*. 2002). The degree of pollen sterility varies depending on the clone, and low seed yield per pollination and seed sterility have frequently been reported as problems in cassava breeding (Hahn *et al*. 1979; Kawano 1980). Studies conducted by Wang *et al.* (2010) revealed that the use of acetocarmine was restricted due to the thick and auto-fluorescent exine wall in cassava. The current study also showed the limitation of using staining methods, such as modified Alexander's staining and the KI method, with mature pollen grains, which could eventually lead to the use of immature flower stages in analyses. The difference between the two staining methods could be attributed to the fact that Alexander's staining allowed for partial staining, which, together with full staining, was considered as viable. On the other hand, with KI staining there were only two classes (stained or unstained). Results indicated that the viability of cassava pollen in immature cyathia (3.2 mm) is high. However, the histological study revealed that pollen viability decreases gradually with the maturity of male cyathia. The reason is unclear and additional studies are required to elucidate the possible reasons for this drastic loss of viability. Attempts to optimize pollen germination in an artificial medium for precise quantification of pollen viability failed as results were inconsistent (data not shown).

Two-size dimorphism in cassava pollen grains was reported by Wang *et al.* (2010); however, a quantitative analysis was not performed. The present study further demonstrates the occurrence of size difference in the lines studied. Results also suggest that this is line dependent, with HMC showing a greater variation in pollen size. Pollen grains with larger or smaller diameters as compared with the normal pollen size were observed, suggesting the existence of pollen trimorphism in cassava. This can be attributed to different reasons. For example, a large pollen size could be attributed to an unreduced pollen grain or dyad condition. The formation of unreduced microspores, also called 2*n* gametes (gametes with somatic chromosome number), appears to be a common phenomenon in angiosperms and could play a major role in polyploid origin and evolution, being reported in cassava (Hahn *et al*. 1990; Vasquez and Nassar 1994; Nelly and Nassar 1995; Nassar 2002). Nassar (1992) reported the formation of polyploids by unreduced gamete fertilization in cassava hybridization. From the plant breeding viewpoint, these gametes are important because they may lead to the development of highly productive triploids and tetraploids by sexual reproduction. They may also play an important role in preserving heterozygosity (Mendiburu and Peloquin 1977). The most direct method of screening for 2*n* pollen involves examining the size range of pollen produced by an individual (Bretagnolle and Thompson 1995). It is well known that cell volume increases with increasing DNA content, which may, in turn, influence pollen diameter (Jansen and Den Nijs 1993). The presence of giant grains has frequently been used as an indicator of 2*n* pollen (Veronesi *et al.* 1988). Based on this information, it was initially hypothesized that dimorphism in cassava could be due to unreduced pollen grains. Study results clearly indicated that large pollen grains are more common in lines SM and TMS. The presence of smaller grains was also observed in the present study, suggesting that the unreduced pollen grains may not be the only cause of dimorphism as reported earlier. The callose matrices containing 8 or 12 cells could be a possible result of abnormal meiosis or mitosis. The majority of these pollen grains, however, were non-viable and it would be interesting to analyse whether the few viable small pollen grains become non-viable later in their development. Of the three lines, HMC presented the lowest viability and highest amount of small pollen grains, clearly indicating a negative correlation between viability and frequency of small pollen grains. Dufay *et al*. (2008) estimated the variation in pollen viability among plants based on pollen size, which was proposed as a good estimator of pollen grain viability.

Histological studies demonstrate that gradual pollen death occurs with maturity, resulting in decreased viability at the time of anthesis. The death of pollen grains was similar in the lines studied after a certain stage of food reserve accumulation. Although viability tests revealed a high percentage (≥80 %) of viable pollen at the tested stage (3.2 mm), it decreased to ∼10 % at anthesis. Liu *et al*. (2007) attributed pollen abortion in *Jatropha* to abnormal development at the tetrad or early-pollen developmental stages*.* Johri (1984) described pollen abortion only before the formation of the tetrads in different dicotyledons. Pollen abortion has also been observed in other Euphorbiaceae such as *Euphorbia pulcherima* (Ai *et al.* 1995) and *Euphorbia dulcis* (Kapil 1961).

Furthermore, a sequential event occurring at the cellular level in the gametogenesis pathway could be compared in the three tested lines. All lines followed an almost similar gametogenesis pathway. Male reproductive development was comparable with that found by Wang *et al*. (2010). The minor differences observed could be attributable to genotype or environmental factors. The different stages of tetrads are the first reported for cassava. These characteristic features will be helpful to identify tetrad maturity when used for microspore culture. The uneven periphery of the mature microspores in tetrads is a unique feature of cassava microsporogenesis prepared for ballooning cell volume once released from the callose matrix. The correlation of the microspore development stage to its relevant diameter would be very useful, whereas the correlation of the development stages to bud size would serve as an external marker for the required bud selection for microspore embryogenesis in the doubled haploid technique. In *Jatropha curcas,* Liu *et al*. (2007) reported that microspores are released into the anther locule by the dissolution of the callose walls, which is comparable with what was observed in the present study. Baker and Baker (1979) reported that all angiosperm pollen grains contain stored food reserves and emphasized the necessity of using only mature pollen to test for starch content as even starchless pollen may accumulate starch at premature stages of development. Starchy pollen has generally been considered a feature of wind-pollinated (anemophilous) flowering plants, whereas insect-pollinated (entomophilous) species show replacement of starch by sugar or lipids (Baker and Baker 1979). The starch reserves stored during pollen development give rise to carbohydrates at maturity, and combinations of different types of carbohydrates in mature pollen may depend on the extent of starch hydrolysis (Speranza *et al*. 1997). Starch accumulation in cassava was also reported by Wang *et al*. (2010). However, the gradual accumulation of protein in the developing pollen grains is reported for the first time in the present study.

To complete the basic demonstration of floral development in cassava, a histological analysis of female gametogenesis was also conducted to provide a basic understanding of sequential events in female gametophyte development. Ovaries exhibited three locular carpels, each carpel containing a single anatropous, bitegmic and crassinucellar ovule. Cassava is monosporic (the most common type of development in Angiosperms), and embryo sac development follows a standard polygonum-type pattern of megasporogenesis. Ogburia and Adachi (1996) provided a histological illustration of a mature embryo sac for identifying meiotic diplospory in cassava; however, no comprehensive illustrations of megasporogenesis were provided.

Cassava ovules showed the unique feature of elaiosomes, a hood-like structure formed by the outer integument and the nucellar beak (an outgrowth of the nucellus), which are common in some members of the Euphorbiaceae family. However, the function and importance of these structures are unclear.

With the formation of the embryo sac, the adjacent nucellus cells degenerated, allowing the expansion of the embryo sac. Takhtadzhian (1991) also reported that most of the nucellus degenerates before the embryo sac reaches maturity. From the beginning of embryo sac formation, signs of such degradation were observed in neighbouring nucellar cells as reported by other researchers (Rodriguez-Rian *et al.* 2006). Starch accumulation in the nucellus just after complete embryo sac formation indicates its role in embryo sac nutrition. Rodriguez-Rian *et al.* (2006) also observed the presence of starch grains in nucellar, outer integument and chalazal cells of the ovule. They also reported the presence of starch grains in central and egg cells, and their greater abundance surrounding the nuclei of both types of cells, with those of the central cell being larger. This seems to indicate that nucellar degradation may supply raw material and/or energy for the accumulation of starch grains in the embryo sac (Rodriguez-Rian *et al.* 2006). A single vascular strand usually runs through the funiculus from the placenta, terminating at the base of the embryo sac—a common feature in most angiosperms, although in some families (including legumes) vascular penetration into one or both integuments has also been observed (Johri *et al*. 1992).

## Conclusions and forward look

This is the first comprehensive report on reproductive development in cassava. Study results indicate that the three lines studied did not differ significantly, except regarding a few morphological aspects such as plant height, flower colour and number of male cyathia. Pollen viability decreased drastically at later developmental stages of pollen grains. Pollen trimorphism is reported in cassava for the first time. Triggering of the pollen trimorphism via abnormal meiosis or mitosis is still to be investigated. The demonstrated sequential events of reproductive development generated valuable information at the cellular level, which will help to close the current information gap in cassava improvement via breeding programmes and doubled haploid plant production.

## Sources of funding

This work was funded by the Bill & Melinda Gates Foundation (USA) through the Grant ID no. OPPGD1483.

## Contributions by the authors

P.I.P.P. was involved in designing the experiments of this research, instructing the methodologies to be used under each experiment, data handling, manuscript preparation and submission. M.Q. provided technical assistance for conducting the experiments. B.D. overlooked and coordinated the project with critical comments. J.D.J.S.K. performed statistical analysis. H.C. contributed certain information about cassava.

## Conflict of interest statement

None declared.
